# The effectiveness of nonsurgical nasal devices in relieving nasal obstruction

**DOI:** 10.1308/rcsann.2025.0103

**Published:** 2025-11-26

**Authors:** A Ahmed, C Gakpetor, D Yang, B Oremule, B Ranganathan, A Jaiswal, RK Bhalla

**Affiliations:** ^1^ Manchester University NHS Foundation Trust, UK; ^2^ Imperial College Healthcare NHS Trust, UK; ^3^ Northern Care Alliance, UK

**Keywords:** Nasal obstruction, Nasal devices, Nasal valve, Nonsurgical devices

## Abstract

**Introduction:**

Nasal obstruction secondary to septal deformity is a common problem, for which surgery is the gold-standard treatment. Long waiting times for surgery, however, leave patients in need of effective, patient-led strategies in the interim. Many wearable nasal devices are commercially available for patients to help relieve nasal obstruction while waiting for surgery; however, the literature around their efficacy is limited.

**Methods:**

A scoping review of the literature was conducted using Medline, Embase, PubMed and Cochrane Library. This was in accordance with the 2018 PRISMA (Preferred Reporting Items for Systematic reviews and Meta Analyses) extension for scoping reviews (PRISMA-ScR).

**Results:**

A total of 186 records were identified from the search, of which 13 met the inclusion criteria. Most studies assessed external nasal devices, in particular the Breathe Right nasal strips, with fewer assessing internal nasal devices. Both devices appear to relieve nasal obstruction; however, objective and subjective outcomes demonstrated greater relief with internal nasal devices. Data on long-term effects were limited.

**Conclusions:**

Nasal valve devices, in particular internal nasal devices, appear to be an effective and accessible method of relieving nasal obstruction. In patients struggling with symptoms and awaiting surgery, these devices could be recommended by clinicians to offer relief in the interim. Further studies on emerging devices such as magnetic nasal strips, which theoretically could be more comfortable to wear, are needed to allow clinicians to fully counsel patients on potential options for treatment.

## Introduction

Nasal obstruction is a common condition presenting to Ear, Nose and Throat (ENT) surgeons, affecting up to 30% of the UK population.^1^ It is well established that nasal obstruction can significantly impact quality of life.^2^

It has been shown that surgery is superior to medical management in treating nasal obstruction secondary to septal deformity.^3^ Many patients’ access to surgery can be significantly delayed, however, with the average waiting time for an initial NHS appointment to see an ENT specialist potentially exceeding 53.3 weeks.^4^ Patients may face a further wait of up to and over 23 weeks once listed for surgery.^5^

Given the above, there is a clear scope to explore alternative, patient-led management strategies.

### Anatomical basis for nasal valve devices

The term nasal valve was first conceptualised in 1903 by Mink to describe the area of maximal resistance of nasal airflow between the caudal portion of the superior lateral cartilage and the nasal septum.^6^ Its clinical significance, however, has been debated in the literature.^7–10^ In 2000, Shaida and Kenyon distinguished between two separate structures: the internal nasal valve, located at the isthmus nasi and influenced by mucosa of the inferior turbinate; and the external nasal valve, located at the nostril rim where airflow is modulated by movement of the inferior lateral cartilage.^10^ Therefore, the evolution from a single valve concept to a dual-valve anatomical model has directly influenced the categorisation and mechanism of nasal devices.

This anatomical distinction has laid the foundation for the development of nonsurgical, wearable devices aimed at alleviating nasal obstruction. A variety of internal and external devices have since been developed to target both the internal and external nasal valve, with the aim of improving symptoms of nasal obstruction.

### Measures of nasal obstruction

A variety of subjective and objective outcomes tools for measuring nasal obstruction have been developed^11^; however, the literature investigating the correlation between the two is heterogeneous.^11^

### Objective measures

In the late 19th and early 20th centuries, Kayser *et al* employed aerodynamic concepts to develop rhinomanometry,^12^ a noninvasive method that measures the pressure changes and corresponding air flow through the nasal cavity.^13^ It is considered the most adequate technique for measuring nasal airflow resistance.^14^

Rhinomanometry can be categorised into both anterior and posterior. Anterior rhinomanometry serves purpose in identifying asymmetrical nasal airflow resistance,^15^ differentiating between anatomical and mucosal irregularities and monitoring treatment response.^16^ Posterior rhinomanometry allows the measurement of nasal airflow in realistic physiological conditions, as well as forceful conditions.^17,18^

Acoustic rhinometry was developed in 1989 as another objective assessment of nasal patency.^19^ It distributes sound waves to calculate cross-sectional areas and volumes of the nasal cavity.^11^ It has been found to be quick, reliable, noninvasive and reproducible.^20^

Another objective measure is Peak Nasal Inspiratory Flow (PNIF), which measures the maximum airflow produced during forced nasal inspiration. It has use in assessing changes in nasal patency,^21^ while being a cheap and portable technique to assess nasal obstruction, with some studies considering it as powerful a tool as rhinomanometry.^22^

Although objective measures of nasal airflow resistance can serve as a useful tool for research purposes,^16^ they are less commonly seen in routine ENT practice.

### Subjective measures

Many subjective assessments of nasal obstruction exist, including visual analogue scales (VAS) or validated questionnaires,^23^ such as the Nasal Obstruction Severity Evaluation (NOSE) score.^24^

Multiple studies have evaluated the relationship between subjective and objective measures of nasal obstruction,^23,25^ and there remains an established but poorly understood phenomenon that subjective perception and objective measures of nasal obstruction are frequently disproportionate.^14^ Given the difficulty in understanding which changes to the nasal airway will translate to improved patient perception of symptoms, a multimodal evaluation of nasal devices is crucial.

This scoping review aims to examine the effectiveness of readily accessible, wearable devices that may be potentially used by patients while awaiting surgery.

## Methods

### Protocol and registration

The scoping review has been reported in accordance with the 2018 PRISMA (Preferred Reporting Items for Systematic reviews and Meta Analyses) extension for scoping reviews (PRISMA-ScR),^26^ and has been registered on the Open Science Framework (OSF): https://osf.io/65puc.

### Eligibility criteria

Primary clinical studies using nonsurgical nasal devices to measure the effect on nasal obstruction on adult patients were included. Patients who were either healthy or had known nasal obstruction were included. Studies involving children (age <18 years), animals, and those exploring surgical or medical management were excluded.

### Information sources and search strategy

**Figure 1:**
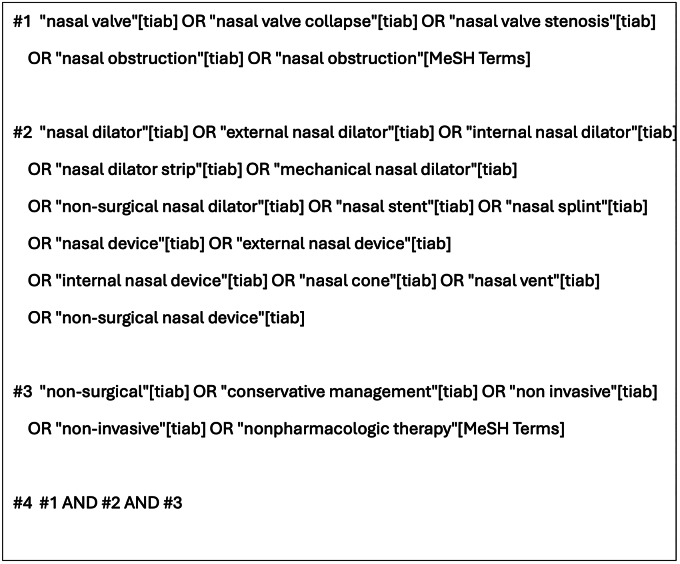
Search terms for Cochrane library

Three reviewers (AA, CG and NG) performed the literature searches independently. MEDLINE, Embase, PubMed and Cochrane Library were used as databases. The search terms used are displayed in Figure 1. There was no limit placed on year of publication; however, a limit was placed for English language only. Two reviewers (AA and CG) then screened abstracts independently.

### Selection of sources of evidence

Two reviewers (AA and CG) independently screened the identified studies. Studies measuring the effect of nonsurgical nasal devices on nasal obstruction were assessed in accordance with the eligibility criteria.

Discrepancies were discussed and resolved with two further reviewers (DY and BO). Studies investigating nonsurgical nasal devices for which a clinician was required for application were excluded.

Studies which measured outcomes specific to nasal airflow and patency were included. This included objective outcomes such as anterior rhinometry, acoustic rhinometry, posterior rhinomanometry and PNIF. Well-documented subjective outcomes for nasal obstruction were included.

Studies measuring objective outcomes not specific to nasal patency, such as imaging techniques or outcomes for exercise tolerance and sleep-disordered breathing were excluded, since our focus was primarily on nasal obstruction. Subjective outcomes which were not well-documented for nasal obstruction were also excluded, such as sleep questionnaires.

Studies which were deemed appropriate underwent full-text screening by both reviewers (AA and CG) independently before data extraction.

## Results

### Study selection

**Figure 2:**
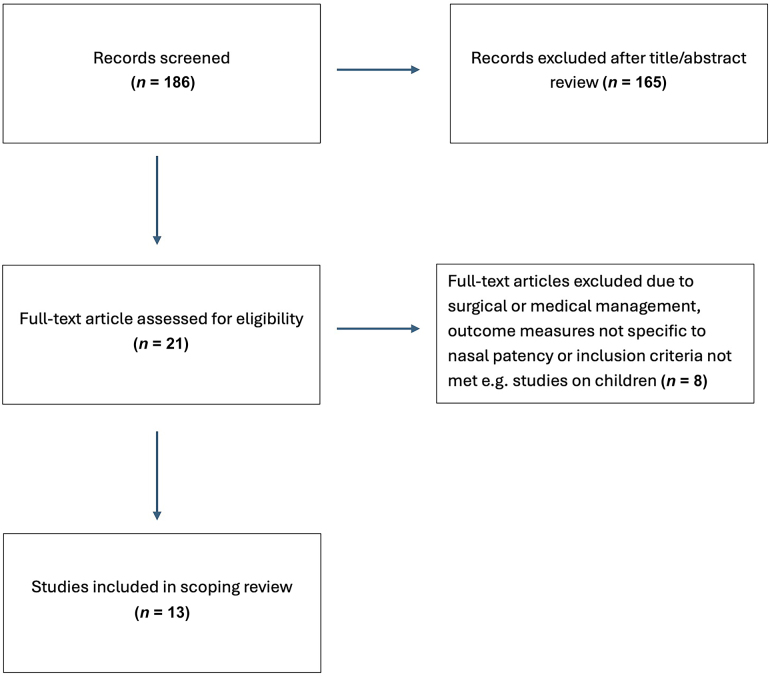
Flowchart of study selection process

A total of 186 records were identified from the literature search for the scoping review. Following screening, 13 records met the inclusion criteria and were included (see Figure 2). Records were then categorised into the type of nasal device, which included internal nasal devices and external nasal devices.

### Study characteristics

The study characteristics, detailing year of publication, study type, number of patients, device type and outcome measured are summarised in Table 1. Overall, ten studies explored external nasal devices and three explored internal nasal devices.

**Table 1:** Study characteristics

Author	Year of publication	Study type	Device type	No. of participants	Outcome measured	Result
J. W. Griffin *et al*^20^	1997	Randomised control trial	External nasal device	53	Acoustic rhinometry	Breathe Right Nasal Strips significantly increased the nasal valve cross-sectional area compared with the placebo. A greater effect was noted among white participants than Black participants; however nil significant difference was found between sexes.
Gosepath *et al*^28^	1997	Prospective observational study with pre-post intervention testing	External nasal device	20	Acoustic rhinometry	Breathe Right Nasal Strips improved transnasal airflow and increased the anterior nasal cavity cross-sectional area particularly at the level of the inferior turbinate.
Lorino *et al*^36^	1997	Prospective experimental study	Internal nasal device	15	Posterior rhinometry	Nozovent (internal nasal dilator), reduced nasal resistance comparably with Pernazene and outperformed Respir (an external nasal dilator)
N. Fergie *et al*^33^	1998	Preliminary study	External nasal device	8	Peak nasal inspiratory flow	All participants reported improved nasal breathing with The Sport Nasal Split. It also increased Peak nasal inspiratory flow values during rest and exercise
Bahammam *et al*^26^	1999	Double-blind randomised control trial	External nasal device	18	Acoustic rhinometry and anterior rhinometry	Breathe Right Nasal strips significantly increased the cross-sectional area of the nasal cavity compared with no device use. They also significantly improved transnasal airflow.
Gosepath *et al*^27^	1999	Prospective observational study, pre-post intervention	External nasal device	26	Anterior rhinomanometry and acoustic rhinometry	Breathe Right Nasal Strips increased intranasal airflow and enlarged the cross-sectional area of the anterior nasal cavity particularly at the level of the inferior turbinate in participants with known nasal obstruction
J.P Kirkness *et al*^29^	2000	Prospective study	External nasal device	20	Posterior rhinometry and nasal patency score	Breathe Right Nasal Strips significantly reduced nasal resistance and improved nasal patency versus the placebo and no device, but less than oxymetazoline. BRNS improved subjective nasal patency even in participants with no objective changes. Breathe Right Nasal Strips and oxymetazoline combined yielded the largest reduction in nasal pressures and patency increase.
L.R. Høyvoll *et al*^14^	2007	Prospective comparative study	External nasal device	89	Nasal symptom scores, acoustic rhinometry and peak inspiratory flow	Breathe Right Nasal Strips increased the cross-sectional area of the nose anteriorly more than xylometazoline and no device use. While it also increased peak inspiratory flow, xylometazoline had a greater effect. However, participants reported a greater subjective increase in nasal patency with Breathe Right Nasal Strips
Brandt *et al*^35^	2008	Prospective randomised control trial	Internal nasal device	23	Posterior rhinometry and western nasal dilation tolerance scale	Internal nasal stents significantly improved nasal airflow more than Breathe Right Nasal Strips and were deemed more comfortable in comparison
Raudenbrush^34^	2011	Randomised control trial	Both	30	Peak nasal inspiratory flow	Max-Air Nose Cones significantly increased peak nasal inspiratory flow more than Breathe Right Nasal Strips in individuals with nasal obstruction
G. Ottaviano *et al*^32^	2017	Double-blind randomised control trial	External nasal device	13	Peak nasal inspiratory flow and NOSE score	Master-aid Roll-Flex increased pre-exercise peak nasal inspiratory flow more than Breathe Right Nasal Strips or no device. However, there was no significant difference in postexercise peak nasal inspiratory flow with or without the devices. Participants reported increased perception of nasal patency with both external nasal dilators.
E.J Schenkel *et al*^31^	2018	Double-blind randomised control trial	External nasal device	59	Nasal breathing VAS 0–100, with 100 representing a clear nose	The Asymmetric Butterfly device and Breathe Right Nasal Strips showed significant subjective increases in nasal patency. Upon removing the strips participants reported longer lasting effect of improved nasal patency with the Butterfly device than Breathe Right Nasal Strips
J.R. Wheatley *et al*^30^	2019	Open-label, uncontrolled exploratory study	External nasal device	48	Acoustic and posterior rhinometry	The Asymmetric Butterfly Prototype significantly increased the nasal cavity area and volume compared with baseline measurements with no device. The protype also reduced the degree of increased nasal resistance when moving from an upright to supine position compared with baseline values without the device

VAS = visual analogue scale.

### Study results

#### External nasal devices

A total of ten studies were identified investigating external nasal devices.^14,20,27–33,35^ The majority focused on the widely known Breathe Right nasal strip (BRNS), with several assessing novel prototypes or ‘sports’ nasal splints.

#### Breathe Right nasal strips

The BRNS comprises two polyester springs covered by adhesive tape^31^ and is applied across the nasal dorsum, superior to the alar cartilages. This lifts the lateral nasal walls to widen the nasal cavity anteriorly.^29^ Six studies assessed the BRNS.^14,20,27–30^ Acoustic rhinometry, performed in most of these studies, consistently demonstrated increases in the total cross-sectional area of the nasal cavity.^20,27–29^

Two studies found that the most notable increase occurred at the level of the inferior turbinate head,^28,29^ with one study reporting an increase as high as 35%.^29^ Improvements in cross-sectional area were found to be similar between men and women^20^; however, one study reported a greater increase in white patients than in Black patients (0.36cm² vs 0.20cm², respectively).^20^ Anterior rhinomanometry displayed increased nasal airflow with BRNS use, with reported improvements ranging from 6% to 16.7%.^28,29^

When BRNS were used in combination with nasal decongestants, an additive effect was observed, with one paper reporting a decrease in nasal airflow resistance of 66%.^30^ When BRNS were compared directly with nasal decongestants based on PNIF values, the latter were the better alternative.^14^ Conversely, subjective assessments using VASs have shown greater perceived nasal airflow improvement with BRNS compared with nasal decongestants.^14^

#### Novel prototype ‘Asymmetric Butterfly’ external nasal device

This novel prototype is similar to the BRNS but adheres to the cheek as opposed to the nose, pulling the lateral nasal wall outwards.^31^ Acoustic rhinometry has shown that it increases cross-sectional area by up to 20% and nasal volume by 22%.^31^ Posterior rhinomanometry has also demonstrated reduced nasal resistance with its use.^31^

Subjective assessments report perceived improvements in breathing, particularly in the supine position^31^; however, when compared with placebo, BRNS were found to be significantly more effective than the novel prototype in subjective assessments.^32^ Nevertheless, the effects of the novel prototype appeared to last longer than the BRNS following device removal as per subjective assessments.^32^

Adverse effects were experienced by some patients wearing this device, ranging from severe pain and application site reactions including bruising and skin abrasions.^31^

#### Master-Aid Roll-Flex strip

The Master-Aid Roll-Flex strip is a customisable adhesive roll of gauze. The gauze can be shaped into pieces to create nasal tip elevation and upper lateral cartilage spread.^33^ Among triathletes, its use led to significantly higher pre-exercise PNIF values compared with no device.^33^

Although effects were comparable with those of BRNS, PNIF values postexercise were found to be similar across all three groups: BRNS, Master-Aid Roll-Flex and no device.^33^ In contrast, subjective assessment using the NOSE score indicated that both devices enhanced perceived nasal airflow postexercise, yet again highlighting the phenomenon that perception and acceptance of nasal blockage may vary,^33^ and thus the importance for future studies to include both subjective and objective measurements of nasal airflow.

#### ‘Sports Nasal Strips’

One study assessed ‘Sports Nasal Strips’, which were described as types of splints containing a strip of plastic with a propensity to straighten, which attach to the upper lateral cartilages via the nasal dorsum.^34^ This in turn dilates the external nasal valve.^34^ The study found that these devices increased PNIF at rest by 19%, and during exercise by 66% in adults.^34^

### Internal nasal devices

A total of three studies were identified investigating internal nasal devices in adults.^35,36,37^ The devices identified were the Max-Air Cones, Nozovent and a novel internal nasal dilator stent.

#### Max-Air nose cones

The Max-Air Cones consist of two soft cones connected by a flexible safety strap that sit inside the nose to dilate the nasal valve.^35^ These devices were assessed by one study only, comparing them directly with the BRNS.^35^ PNIF values in subjects with known nasal obstruction increased by 110% when using Max-Air Cones, compared with 54% when using the BRNS.

#### Novel internal nasal dilator stent

The Novel internal nasal dilator stent comprises steel springs connected by a bridge, which equally apply pressure to open the internal nasal valve.^36^

Subjective assessments using the Western Nasal Dilatation Tolerance Scale (WNDTS)^36^ have shown this novel device to be significantly more comfortable and easier to use when compared with the BRNS, with a significantly higher overall tolerability score.^36^ The mean cumulative wear-time over seven days investigated by one study was 69.43±2.27 hours for the novel device, compared with 43.91±1.56 hours for the BRNS.^36^ Posterior rhinomanometry demonstrated a much more significant reduction in nasal resistance from baseline when using this novel device (3.4 times improved nasal airflow) compared with the BRNS.^36^

#### Nozovent

The Nozovent internal nasal device consists of a plastic bar with tabs on both ends to insert in each nostril.^37^ Posterior rhinomanometry demonstrated significant reductions in nasal resistance, much greater than was shown with an external nasal device.^37^ However, when compared with oxymetazoline, results were comparable, making Nozovent a suitable alternative in those concerned about long-term pharmacological treatment.^37^ This paper highlighted that no discomfort or side effects were reported regarding either treatment.

## Discussion

### Summary of evidence

Our scoping review suggests that both internal and external nasal devices may be fast, effective and simple solutions to alleviate nasal obstruction.

Among external devices, the BRNS was the most extensively studied, producing consistent increases in nasal cross-sectional area and airflow,^20,27–29^ with effects comparable with those of the Master-Aid Roll-Flex strip but superior to those of the novel external asymmetric butterfly prototype. Objective performance was greater in patients with higher resting nasal resistance.^30^ One study also suggested variation in outcomes by ethnicity,^20^ although these findings should be interpreted with caution as ethnicity was not clearly defined and other confounding factors (such as body habitus and athleticism) were not accounted for. Compared with nasal decongestants, BRNS achieved lower PNIF values but were favoured in subjective assessments, offering a safer long-term alternative given the risks of prolonged decongestant use such as cardiovascular and central nervous system complications,^14^ as well as risking rhinitis medicamentosa with long-term usage.

The asymmetric butterfly prototype was the only external device that showed prolonged post-removal effects,^32^ although comfort and skin irritation were limiting factors.^31^

Among studies on internal devices assessing PNIF (Max-Air Cones and Nozovent),^35,37^ the Max-Air cones produced the greatest increase; however, study populations varied, as Max-Air Cones were assessed on people with known nasal obstruction and Nozovent was tested on healthy subjects, limiting direct comparison.

When comparing both internal and external nasal devices, three papers compared them directly: Max-Air Cones versus BRNS,^35^ Novel Internal Nasal Dilator Stent versus BRNS,^36^ and Nozovent versus Respir+.^37^ These suggested that, although external nasal devices are objectively effective in relieving nasal obstruction, their PNIF and posterior rhinomanometry outcomes overall may not match those achieved with internal nasal devices. Evidence from a single study suggested that an internal nasal device was found more comfortable and easier to use than the BRNS.^36^ The significantly greater mean wear-time of an internal nasal device compared with the BRNS (>50%) is a noteworthy finding given the focus of this review^36^: to explore accessible, wearable devices that patients can use independently while awaiting specialist care.

Overall, our review suggests that internal nasal devices may offer superior efficacy in both objective and subjective airflow metrics, perhaps suggesting they are more sustainable for daily use.

### Limitations

Patient populations varied between studies, some of which were not representative of the target population. For example, Gosepath and Høyvoll *et al* recruited only white patients,^14,29^ and it is known that nasal airflow and resistance may vary between ethnicities.^14^ Only one study on external devices separated results between sex and ethnicity (white versus Black patients)^20^; however, ethnicity was not defined by the authors and potential confounders such as body habitus and athletic training were not controlled for. As such, anatomical variability was not fully accounted for, reducing the generalisability of the findings. None of the papers on internal nasal devices specified ethnicity of participants, limiting generalisability; however, most had an equal number of male to female participants. Certain studies recruited only healthy participants, who may not experience similar effects to those with nasal obstruction who require nasal devices.

Another limitation concerns the characterisation of nasal obstruction in the included studies. All recruited patients across the included studies were either healthy or had a history of impaired nasal breathing. None of the studies specified the anatomical level of nasal obstruction, or whether symptoms were attributable to nasal valve collapse or general nasal obstruction. This is a limitation since the outcome of devices may differ depending on the anatomical site of nasal obstruction.

Furthermore, procedural consistency varied between studies. Griffin *et al* and Kirkness *et al* were the only authors to describe having the same person applying the nasal devices to each participant,^20,30^ thus risking interoperator variability and performance bias in the other studies. Similarly, only one study mentioned using the same operator to handle the rhinometer and calibration checks.^14^

Only four studies reported on device comfort, safety and adverse effects,^31,32,36,37^ limiting the ability to understand patient experience. No follow-up of patients was performed, making it difficult to assess the devices’ long-term effects and durability.

Several studies were conducted by device manufacturers or patent holders,^35,36^ introducing the potential for conflict of interest, as well as funding and reporting bias.

Most studies in this review relied on the use of objective measures of nasal obstruction. In the current literature objective improvement does not always correlate with subjective improvement in patients with nasal obstruction. This potentially raises questions regarding the clinical relevance of objective measures, and some studies suggest objective outcomes should be limited to quantifying surgical results only.^25,38^ Given subjective nasal obstruction is multifactorial, future studies should focus on using validated patient-reported outcome measures such as the NOSE score or VASs, to better capture the clinical impact of these devices.

Finally, there has recently been an influx of online advertisements for wearable external magnetic nasal devices for nasal obstruction. During our search for studies, there were no studies available on these devices. There is therefore a clear need for updated studies on the latest devices available.

### Implications for future practice

Both device types, notably internal nasal devices, demonstrated significant improvements in nasal airflow, making them potential treatment options in regular clinical practice. Given increasing waiting times for surgery, these devices could be deployed in primary care or secondary care for the initial ENT appointment. Here, patients could undergo rapid assessment and offered a trial of a device on the same day, enabling symptom relief for those waiting surgery or those unable to undergo surgery. This would contribute towards more patient-led care, especially since these devices do not require clinician assistance, further helping relieve burden on an over-stretched NHS and expediting symptom control in patients.

## Conclusion

Our scoping review suggests that nasal devices, particularly internal nasal devices, are effective and comfortable tools to alleviate nasal obstruction. While surgery remains the gold standard, in the current climate of prolonged waiting times for NHS appointments and surgery, these devices are wearable devices which patients can utilise as an interim measure, or if surgery is not wanted. Clinicians should be aware of all available treatment options for patients with nasal obstruction secondary to septal deviation. Further studies are required to investigate emerging devices, such as external magnetic nasal devices, and establish long-term durability and side effects to appropriately counsel patients.

## Competing interests

The author/s declare no competing interests.

## Funding

The author/s received no financial support for the research, authorship and/or publication of this article.

## Ethics approval and consent to participate

Not applicable.

## Artificial Intelligence

The author/s declare that no AI was used to conduct the study or prepare the manuscript.

## References

[1] Fageeh YA, Basurrah MA, ALAzwari KD *et al.* Prevalence of nasal obstruction and its impact on quality of life in Saudi Arabia. *J Family Med Prim Care* 2024; **13**: 572–578. 38605785 10.4103/jfmpc.jfmpc_482_23 PMC11006041

[2] Li CH, Kaura A, Tan C *et al.* Diagnosing nasal obstruction and its common causes using the nasal acoustic device: a pilot study. *Laryngoscope Investig Otolaryngol* 2020; **5**: 796–806. 10.1002/lio2.445 PMC7461538 32904889

[3] Carrie S, O’Hara J, Fouweather T *et al.* Clinical effectiveness of septoplasty versus medical management for nasal airways obstruction: multicentre, open label, randomised controlled trial. *BMJ* 2023; **383**: e075445. 37852641 10.1136/bmj-2023-075445 PMC10583133

[4] East Sussex Healthcare NHS Trust. Average wait for routine first outpatient appointment. https://www.esht.nhs.uk/average-wait-for-routine-first-outpatient-appointment/ (cited May 2025).

[5] Manchester University NHS Foundation Trust. Ear, nose and throat – my planned care NHS. https://www.myplannedcare.nhs.uk/nwest/manchester/specialty/?sname=Ear,%20Nose%20and%20Throat (cited May 2025).

[6] Mink PJ. *Physiologie der oberen Atemwege: Die Nasenhöhle*. Leipzig: F.C.W. Vogel; 1920.

[7] Bridger GP, Proctor DF. Maximum nasal inspiratory flow and nasal resistance. *Ann Otol Rhinol Laryngol* 1970; **79**: 481–488. 5426879 10.1177/000348947007900308

[8] Bachmann W, Legler U. Studies on the structure and function of the anterior section of the nose by means of luminal impressions. *Acta Otolaryngol* 1972; **73**: 433–442. 5031970 10.3109/00016487209138963

[9] Hirschberg A, Roithmann R, Parikh S *et al.* The airflow resistance profile of healthy nasal cavities. *Rhinology* 1995; **33**: 10–13. 7540313

[10] Shaida AM, Kenyon GS. The nasal valves: changes in anatomy and physiology in normal subjects. *Rhinology* 2000; **38**: 7–12. 10780041

[11] Naito K, Horibe S, Tanabe Y *et al.* Objective assessment of nasal obstruction. *Fujita Med J* 2023; **9**: 53–64. 37234397 10.20407/fmj.2021-029 PMC10206903

[12] Kayser R. Die Exacte Messung der Luftdurchgangigkeit der Nase. *Arch Laryngol* 1895; **3**: 101–120.

[13] Avrunin OG, Nosova YV, Abdelhamid IY *et al.* Research active posterior rhinomanometry tomography method for nasal breathing determining violations. *Sensors (Basel)* 2021; **21**: 8508. 34960601 10.3390/s21248508 PMC8708127

[14] Høyvoll LR, Lunde K, Li HS *et al.* Effects of an external nasal dilator strip (ENDS) compared to xylometazoline nasal spray. *Eur Arch Otorhinolaryngol* 2007; **264**: 1289–1294. 17530269 10.1007/s00405-007-0345-6

[15] Demirbas D, Cingi C, Cakli H, Kaya E. Use of rhinomanometry in common rhinologic disorders. *Expert Rev Med Devices* 2011; **8**: 769–777. 22029472 10.1586/erd.11.45

[16] Patil N, Jain S. Rhinomanometry: a comprehensive review of its applications and advancements in rhinology practice. *Cureus* 2024; **16**: e61370. 38947630 10.7759/cureus.61370 PMC11214531

[17] Selivanova KG, Avrunin OG, Zlepko SM *et al.* *Quality improvement of diagnosis of the electromyography data based on statistical characteristics of the measured signals*. In: Proc. SPIE 10031, Photonics Applications in Astronomy, Communications, Industry, and High-Energy Physics Experiments 2016, 100312R (28 September 2016). Bellingham (WA): SPIE, 2016.

[18] Avrunin OG. Improving the reliability of rhinomanometry diagnostics by considering statistical characteristics of measured signals. *Telecommun Radio Eng* 2014; **73**: 647–655.

[19] Grymer LF, Hilberg O, Elbrønd O, Pedersen OF. Acoustic rhinometry: evaluation of the nasal cavity with septal deviations, before and after septoplasty. *Laryngoscope* 1989; **99**: 1180–1187. 2682101 10.1288/00005537-198911000-00015

[20] Griffin JW, Hunter G, Ferguson D, Sillers MJ. Physiologic effects of an external nasal dilator. *Laryngoscope* 1997; **107**: 1235–1238. 9292609 10.1097/00005537-199709000-00014

[21] Fornadley JA. The stuffy nose and rhinitis. *Med Clin North Am* 1999; **83**: 1–12. 9927956 10.1016/s0025-7125(05)70083-3

[22] Ottaviano G, Lund VJ, Nardello E *et al.* Comparison between unilateral PNIF and rhinomanometry in healthy and obstructed noses. *Rhinology* 2014; **52**: 25–30. 24618624 10.4193/Rhino13.037

[23] Mozzanica F, Gera R, Bulgheroni C *et al.* Correlation between objective and subjective assessment of nasal patency. *Iran J Otorhinolaryngol* 2016; **28**: 313–319. 27738607 PMC5045701

[24] Stewart MG, Witsell DL, Smith TL *et al.* Development and validation of the nasal obstruction symptom evaluation (NOSE) scale. *Otolaryngol Head Neck Surg* 2004; **130**: 157–163. 14990910 10.1016/j.otohns.2003.09.016

[25] Umihanic S, Brkic F, Osmic M *et al.* The discrepancy between subjective and objective findings after septoplasty. *Med Arch* 2016; **70**: 336–338. 27994291 10.5455/medarh.2016.70.336-338 PMC5136432

[26] Tricco AC, Lillie E, Zarin W, *et al.* PRISMA extension for scoping reviews (PRISMA-ScR): checklist and explanation. *Ann Intern Med* 2018; **169**: 467–473. 30178033 10.7326/M18-0850

[27] Bahammam AS, Tate R, Manfreda J, Kryger MH. Upper airway resistance syndrome: effect of nasal dilation, sleep stage, and sleep position. *Sleep* 1999; **22**: 592–598. 10450594

[28] Gosepath J, Amedee RG, Romantschuck S, Mann WJ. Breathe right nasal strips and the respiratory disturbance index in sleep-related breathing disorders. *Ear Nose Throat J (Burlington)* 1999; **78**: 544–548. 10.2500/105065899781367456 10582117

[29] Gosepath J, Mann WJ, Amedee RG. Effects of the breathe right nasal strips on nasal ventilation. *Am J Rhinol* 1997; **11**: 399–402. 9768323 10.2500/105065897781285990

[30] Kirkness JP, Wheatley JR, Amis TC. Nasal airflow dynamics: mechanisms and responses associated with an external nasal dilator strip. *Eur Respir J* 2000; **15**: 929–936. 10853861 10.1034/j.1399-3003.2000.15e20.x

[31] Wheatley JR, Amis TC, Lee SA *et al.* Objective and subjective effects of a prototype nasal dilator strip on sleep in subjects with chronic nocturnal nasal congestion. *Adv Ther* 2019; **36**: 1657–1671. 31119695 10.1007/s12325-019-00980-z PMC6822853

[32] Schenkel EJ, Ciesla R, Shanga GM. Effects of nasal dilator strips on subjective measures of sleep in subjects with chronic nocturnal nasal congestion: a randomized, placebo-controlled trial. *Allergy Asthma Clin Immunol* 2018; **14**: 34. 30154874 10.1186/s13223-018-0258-5 PMC6109978

[33] Ottaviano G, Ermolao A, Nardello E *et al.* Breathing parameters associated to two different external nasal dilator strips in endurance athletes. *Auris Nasus Larynx* 2017; **44**: 713–718. 28153693 10.1016/j.anl.2017.01.006

[34] Fergie N, Bingham BJG. Do sports nasal strips improve nasal airflow? A preliminary report. *J Otolaryngol* 1998; **27**: 113–116. 9572466

[35] Raudenbush B. Stenting the nasal airway for maximizing inspiratory airflow: internal max-air nose cones versus external breathe right strip. *Am J Rhinol Allergy* 2011; **25**: 249–251. 21819762 10.2500/ajra.2011.25.3621

[36] Brandt MG, Moore CC, Doyle PC. Clinical evaluation of a novel internal nasal dilation stent for the improvement of nasal breathing. *Otolaryngol Head Neck Surg* 2008; **138**: 626–632. 18439469 10.1016/j.otohns.2008.01.007

[37] Lorino AM, Lofaso F, Drogou I *et al.* Effects of different mechanical treatments on nasal resistance assessed by rhinometry. *Chest* 1998; **114**: 166–170. 9674465 10.1378/chest.114.1.166

[38] André RF, Vuyk HD, Ahmed A *et al.* Correlation between subjective and objective evaluation of the nasal airway: a systematic review of the highest level of evidence. *Clin Otolaryngol* 2009; **34**: 518–525. 20070760 10.1111/j.1749-4486.2009.02042.x

